# Noninvasive prediction of TP53 mutation in prostate cancer based on advanced diffusion weighted imaging

**DOI:** 10.3389/fonc.2026.1854578

**Published:** 2026-06-22

**Authors:** Juan Chen, Han-Xi Zhang, Xian-Wen Cheng, Jie Bian, Hua-Bin Huang, Shu-Yi Li, Hui-Ling Xiao, Di-Min Liu, Kun-Peng Zhou

**Affiliations:** 1 Medical Imaging Center, Shenzhen Pingle Orthopedic Hospital (Shenzhen Pingshan Traditional Chinese Medicine Hospital), Shenzhen, China; 2 Department of Radiology, The Seventh Affiliated Hospital, Sun Yat-sen University, Shenzhen, China; 3 Department of Radiology, Second Affiliated Hospital of Dalian Medical University, Dalian, China

**Keywords:** diffusion kurtosis imaging, imaging biomarker, prostate cancer, stretched exponential model, TP53

## Abstract

**Background:**

TP53 mutation is associated with poor prognosis and resistance to androgen deprivation therapy in prostate cancer (PCa). This study investigated whether stretched exponential model (SEM) and diffusion kurtosis imaging (DKI) could serve as a non-invasive predictor of TP53 mutation.

**Methods:**

This retrospective study included 84 patients with PCa who underwent radical prostatectomy. Clinical, clinicopathological, and quantitative imaging parameters were compared between the two groups using the independent-samples t test, chi-square test, or Fisher’s exact test, as appropriate. Univariate and multivariate binary logistic regression analyses were performed to identify factors associated with TP53 mutation. Five logistic regression models were constructed: model 1 (based on DKI), model 2 (based on SEM), model 3 (based on mono-exponential model), model 4 (based on DKI, SEM and mono-exponential model) and model 5 (based on mean kurtosis (MK) value). Receiver operating characteristic analysis, DeLong test, Akaike information criterion (AIC), decision curve analysis (DCA), calibration curves, Hosmer–Lemeshow test, and bootstrap internal validation were used to evaluate model performance.

**Results:**

Compared with the TP53 wild-type group, the TP53 mutated group showed significantly lower apparent diffusion coefficient (ADC), distributed diffusion coefficient (DDC), and mean diffusivity (MD) values and a significantly higher MK value (all *p* < 0.05). In univariate logistic regression, MK, MD, DDC, and ADC values were significantly associated with TP53 mutation (all *p* < 0.05). In multivariate analysis, only MK value (OR = 19.329, *p* = 0.004) remained independent predictors. Among the four models, Model 4 achieved the highest AUC (0.889, 95% CI: 0.821–0.958), followed by Model 1 (0.881, 95% CI: 0.811–0.952) and Model 5 (0.860, 95% CI: 0.784–0.937). However, no significant difference was observed among Models 1, 4 and 5 (all *p*>0.05). The AIC of model 5 was 79.739, which was well calibrated. The net benefit of DCA was robust, and the AUC of optimistic correction was 0.858, which indicated good internal stability.

**Conclusions:**

MK value is an independent predictor of TP53 mutation in PCa. MK value-based model demonstrated good diagnostic performance and outperformed the SEM and mono-exponential model, suggesting that MK value may serve as a promising preoperative, noninvasive tool for assessing TP53 mutation status in PCa.

## Introduction

Prostate cancer (PCa) is one of the most common malignancies in men and the fifth leading cause of cancer-related death worldwide (1). Patients with localized or locally advanced PCa generally have a favorable prognosis, whereas those with metastatic disease—particularly castration-resistant prostate cancer (CRPC)—experience poor outcomes, with a median survival typically of less than two years (2, 3). For advanced PCa, androgen deprivation therapy (ADT) targeting the androgen receptor (AR) signaling pathway remains one of the main therapeutic strategies (4).

Although most patients achieve a marked reduction in tumor burden during the initial phase of ADT, nearly all eventually develop resistance to AR inhibition and progress to CRPC. The majority of CRPCs evade AR-directed therapy by directly or indirectly reactivating AR signaling; however, approximately 15–20% of CRPCs lose their dependence on AR and evolve into neuroendocrine prostate cancer (NEPC), which carries an even worse prognosis (5, 6). Increasing evidence suggests that, under chronic androgen-deprived pressure, loss of TP53 (frequently co-occurring with RB1 loss) attenuates AR signaling and reprograms multiple metabolic pathways, including glucose–lactate, glycogen, and lipid metabolism. These alterations confer enhanced metabolic plasticity and stem-like properties, thereby driving the transition from androgen-sensitive adenocarcinoma to CRPC and ultimately to NEPC (6, 7).

TP53 mutation is among the most frequent genetic alterations in PCa, occurring in approximately 50% of metastatic cases and often co-existing with other key genomic events (8). Previous studies have demonstrated that TP53 loss represents a critical prognostic biomarker in PCa and is associated with resistance to novel hormonal agents such as abiraterone and enzalutamide (9, 10). Consequently, accurate assessment of TP53 status is crucial for optimizing treatment strategies and improving outcomes in metastatic PCa. However, current evaluation relies predominantly on histopathologic and molecular assays, which are invasive and not well suited for dynamic monitoring.

The stretched exponential model (SEM) and diffusion kurtosis imaging (DKI) provide insight into tissue microstructural complexity and heterogeneity by quantifying the non-Gaussian diffusion behavior of water molecules in biological tissues (11, 12). Prior studies have shown that SEM and DKI can not only improve the detection of clinically significant PCa (Gleason score ≥ 4 + 3) but also hold promise for assessing tumor aggressiveness (13–16). Specifically, parameter distributed diffusion coefficient (DDC) of SEM and parameter mean diffusivity (MD) of DKI are significantly and inversely correlated with Gleason pattern, whereas parameter mean kurtosis (MK) of DKI shows a significant positive correlation with Gleason pattern (15–17). Nevertheless, whether TP53 mutation status is associated with parameters of SEM and DKI in PCa remains unclear.

In this context, the present study aims to investigate the relationship between parameters of SEM and DKI with TP53 mutation status in PCa, and to determine whether these parameters can serve as non-invasive imaging biomarkers for TP53 mutation.

## Materials and methods

### Patients

**Figure 1:**
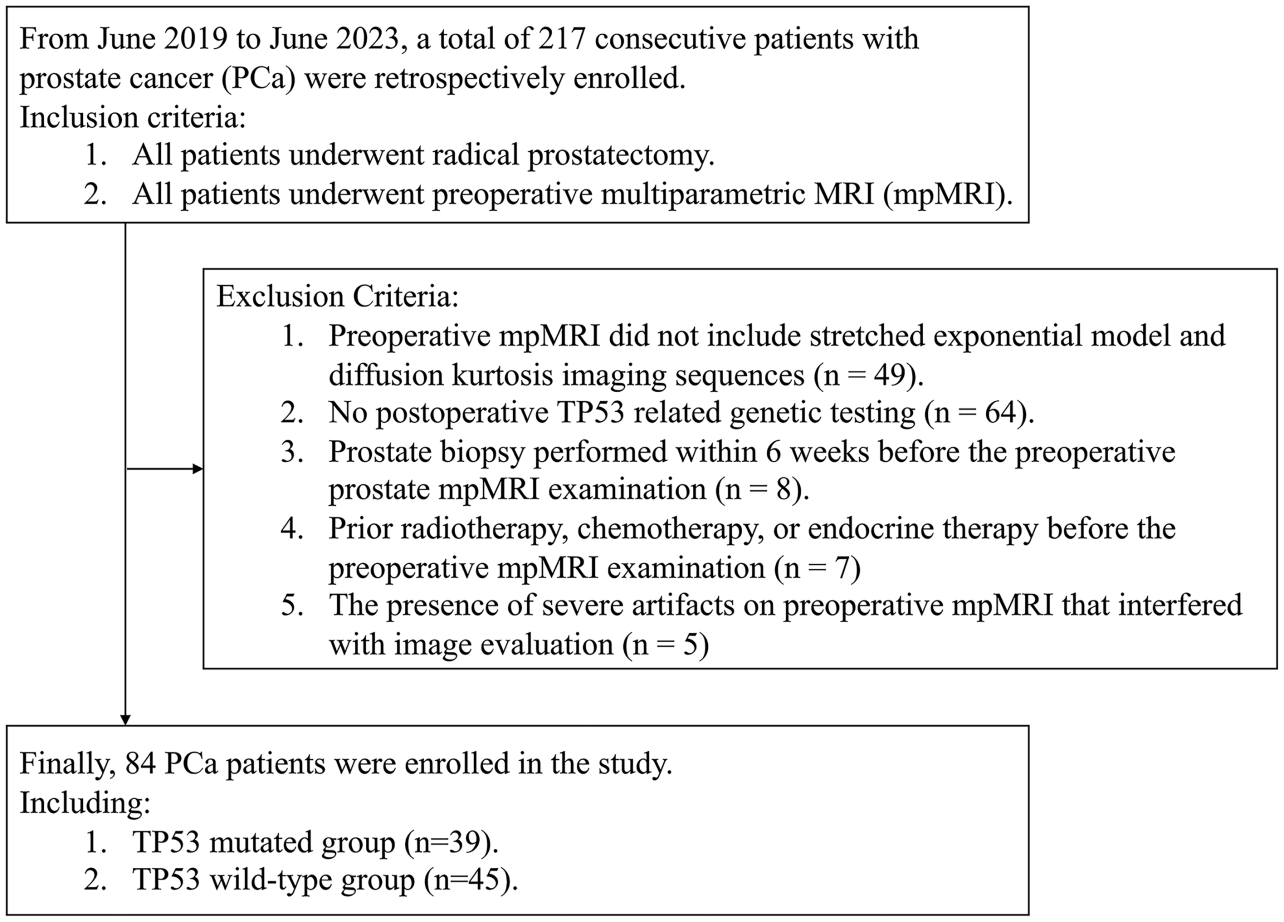
Patient flow chart.

This retrospective study was approved by *the Ethics Committee of the Second Hospital of Dalian Medical University* (PR/AG 2023-268, approved on 4 September 2023). From June 2019 to June 2023, a total of 217 consecutive patients with PCa who underwent radical prostatectomy and preoperative multiparametric MRI (mpMRI) at our institution were retrospectively enrolled. The exclusion criteria were as follows: (1) preoperative mpMRI did not include SEM and DKI sequences (n = 49); (2) no postoperative TP53 related genetic testing (n = 64); (3) prostate biopsy performed within 6 weeks before the preoperative prostate mpMRI examination (n = 8); (4) prior radiotherapy, chemotherapy, or endocrine therapy before the preoperative mpMRI examination (n = 7); and (5) the presence of severe artifacts on preoperative mpMRI that interfered with image evaluation (n = 5). Finally, 84 patients with PCa were included in the analysis, of whom 39 were TP53 mutated group and 45 were TP53 wild-type group. The details are shown in **Figure 1**.

### MRI techniques

All MRI examinations were performed on a 3.0-T system (Discovery MR 750W, GE Healthcare) using an 8-channel phased-array coil. The scanning protocol included conventional sequences such as T_2_-weighted imaging (T_2_WI), T_1_-weighted imaging (T_1_WI), diffusion-weighted imaging (DWI), and dynamic contrast-enhanced MRI (DCE-MRI). SEM and DKI, as multi-b-value advanced DWI sequences, the b-value settings of SEM in the study were as follows: eleven b values were used for SEM, and b values [number of excitations] were 0[1], 25[1], 50[1], 75[1], 100[4], 150[4], 200[6], 400[8], 800[10], 1200[12], and 2000[14] s/mm² (18); DKI was acquired with 15 diffusion gradient directions and 5 b values, with b values [number of excitations] of 0[1], 500[2], 1000[4], 1500[6], and 2000[8] s/mm² (19). Detailed sequence parameters were summarized in **Supplementary Material** (**Supplementary Table 1**).

### Quantitative data analysis

All quantitative data measurements were performed on a GE AW 4.6 workstation by a single radiologist (KP.Z., with 8 years of experience in MRI). First, the slice containing the largest cross-sectional area of the tumor was identified on apparent diffusion coefficient (ADC) map, and a region of interest (ROI) was manually drawn along the tumor outline on this slice. Subsequently, the slices at which the maximum cross-sectional area of the tumor was located were determined on the corresponding parametric maps of SEM and DKI. Finally, the ROI delineated on the ADC map was then copied to the corresponding SEM- and DKI-related parametric maps. The mean values of the lesion-related parameters within the ROI were measured and recorded for subsequent statistical analysis. All ROIs were drawn to avoid intralesional cystic degeneration, necrosis, hemorrhage, and the urethra.

### Histopathology analysis

All patients underwent radical prostatectomy performed by experienced urologists. Immediately after surgery, the resected prostate specimens containing PCa lesions were fixed in formalin, embedded in paraffin, and serially sectioned from the apex to the base at 3-mm intervals. All sections were stained with hematoxylin and eosin (H&E) and subsequently subjected to histopathological evaluation. To achieve the closest possible correspondence between the imaging slice used for quantitative analysis (i.e., the slice on which the ROI was delineated) and the histopathological evaluation slice, the following procedure was used to assess TP53 mutation status. First, the slice showing the largest cross-sectional area of the lesion was identified on axial ADC map, and the ROI was delineated on this slice. Next, the vertical distance from this slice to the prostatic apex was measured on sagittal T_2_WI and recorded as D (mm). Based on the measured distance D and the 3-mm interval between histopathological sections, the corresponding histological section number was estimated to identify the most appropriate section for TP53 mutation status assessment. Multiple sampling points were then selected on the identified histological section for evaluation of TP53 mutation status.

TP53 mutation status was determined as follows: genomic DNA was extracted from formalin-fixed, paraffin-embedded postoperative tissue sections using a commercial DNA extraction kit, followed by mutation analysis of the TP53 gene via polymerase chain reaction. Based on the results, patients were categorized as TP53 mutated group and wild-type group. Histopathological evaluation was performed by a single pathologist (K.G.) with over 10 years of diagnostic experience. All histopathological assessments were reported in accordance with the International Society of Urological Pathology (ISUP) guidelines (20).

### Statistical analysis

Statistical analyses were performed using Python (version 3.13). A two-tailed *p* value < 0.05 was considered statistically significant. Continuous variables were presented as mean ± standard deviation, and categorical variables were expressed as frequencies and percentages. Differences between the TP53 wild-type and mutated groups were compared using the independent-samples *t* test for continuous variables and the chi-square test or Fisher’s exact test for categorical variables, as appropriate. Univariate binary logistic regression analysis was performed to assess the associations between TP53 mutation and clinicopathological and quantitative imaging variables. Prior to performing multivariate binary logistic regression analysis, the variance inflation factor (VIF) was employed to test for multicollinearity among the variables. Variables free of multicollinearity were subsequently incorporated into the multivariate binary logistic regression analysis to identify independent predictors of TP53 mutation. Five prediction models were further established using logistic regression: Model 1 based on DKI (MK and MD values), Model 2 based on SEM (DDC and heterogeneity index (α) values), Model 3 based on mono-exponential model (ADC value), Model 4 based on DKI, SEM and mono-exponential model and Model 5 based on MK value. Their diagnostic performance was evaluated using ROC curve analysis, and the AUCs were compared using the DeLong test. AIC was used to compare model fit, with lower values indicating better model performance. DCA was performed to compare the clinical net benefit of the four models across a range of threshold probabilities. Calibration was assessed using calibration curves and the Hosmer–Lemeshow test. Internal validation of the optimal model was conducted using bootstrap resampling (1,000 iterations), and the optimism-corrected AUC, Brier score, calibration intercept, and calibration slope were calculated.

## Results

### Clinical characteristics

**Figure 2:**
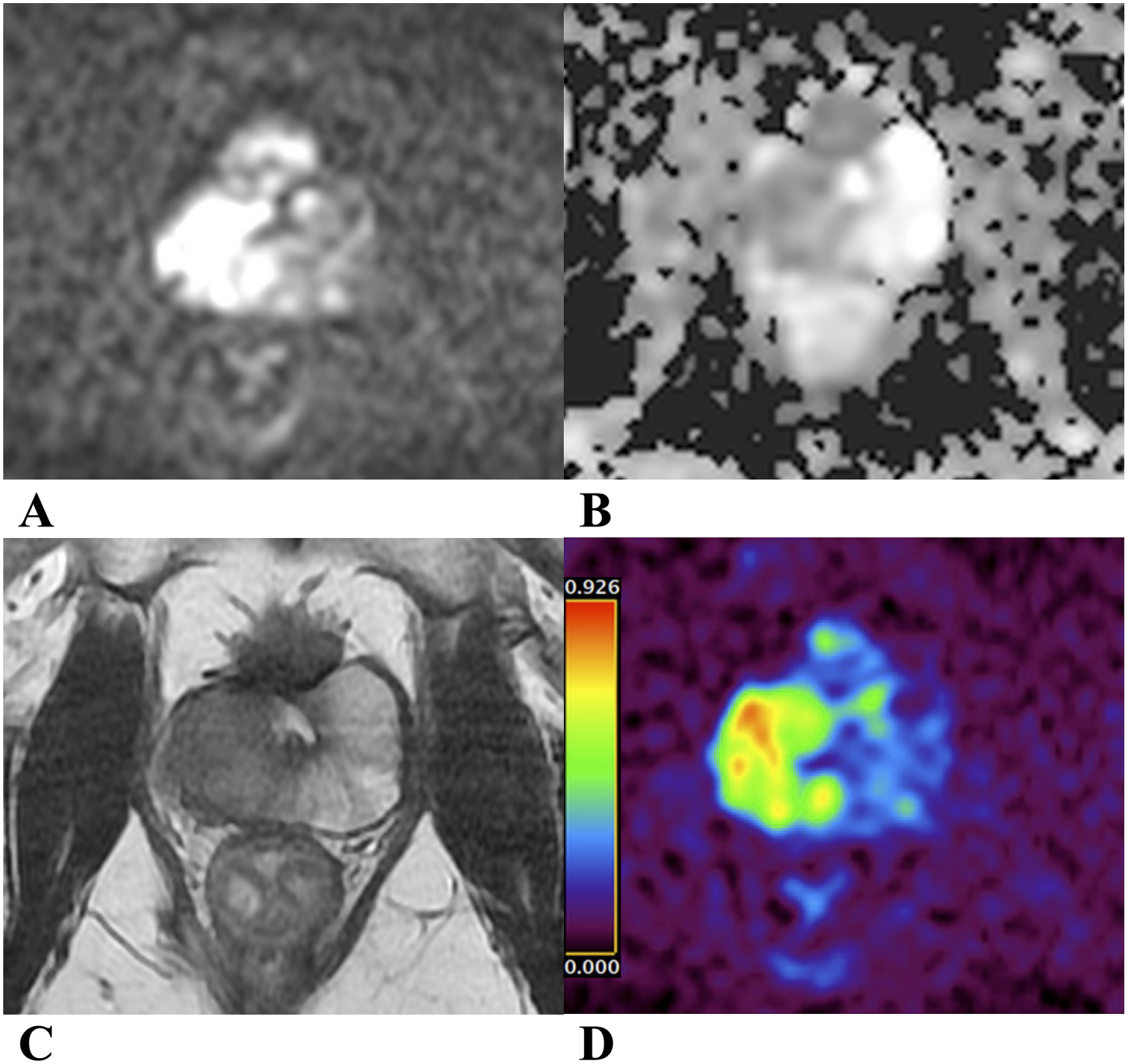
A 73-year-old TP53 mutated group prostate cancer patient, prognostic grade group was 4, T-PSA was 15.18 ng/ml, F-PSA was 2.37 ng/ml, baseline F/T-PSA was 0.16. Preoperative multi-parametric MRI [**(A)** diffusion weighted imaging; **(B)** apparent diffusion coefficient map; **(C)** T_2_ weighted imaging] showed that the PI-RADS 5 lesion was located in the right peripheral zone, and the mean kurtosis (MK) value was 0.685; **(D)** Color-coded parametric map showing the lesion, with a color scale bar ranging from 0 to 0.926.

**Figure 3:**
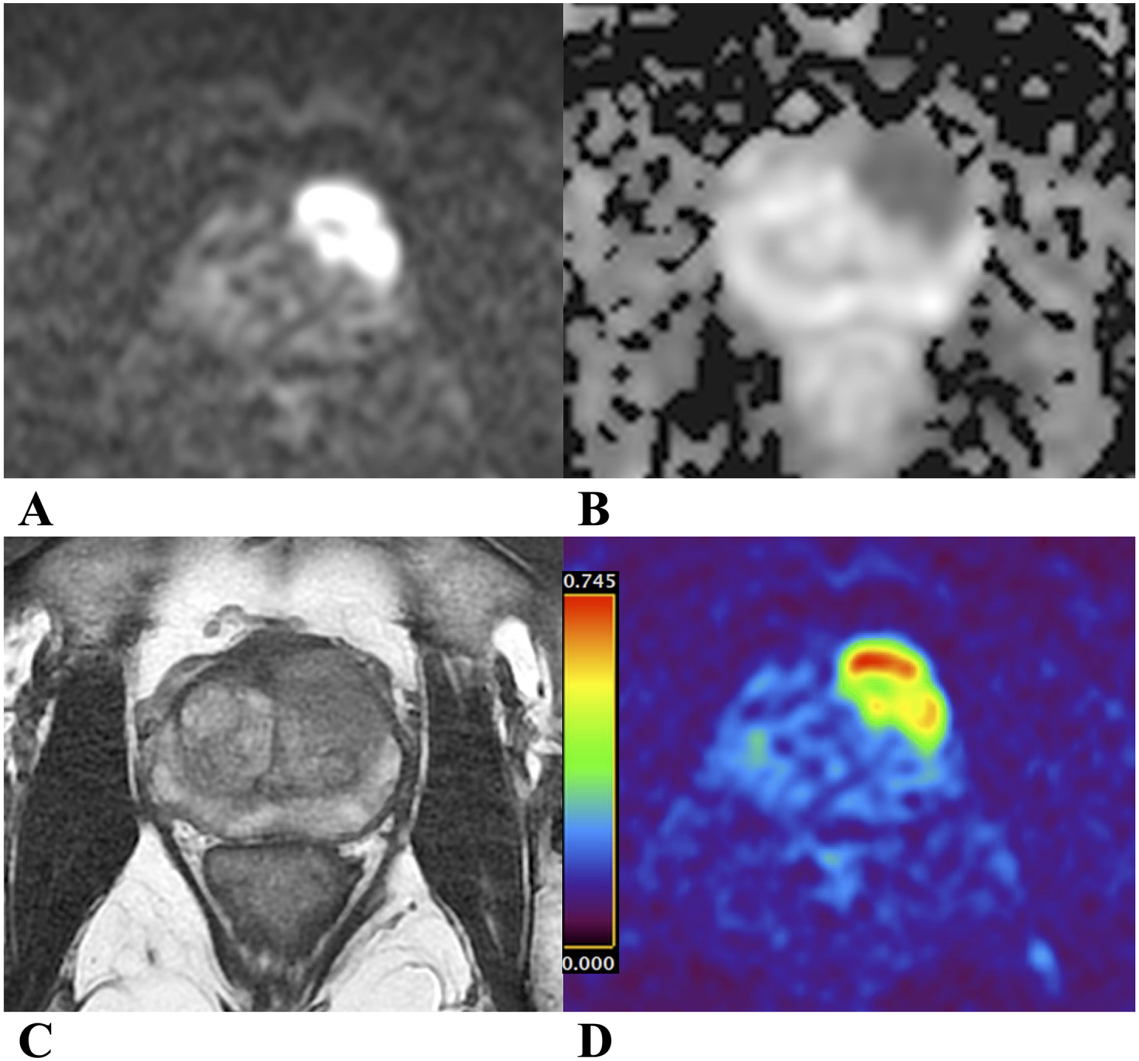
A 76-year-old TP53 wild-type group prostate cancer patient, prognostic grade group 3, T-PSA was 13.93 ng/ml, baseline F-PSA was 2.87 ng/ml, baseline F/T-PSA was 0.21. Preoperative multi-parametric MRI [**(A)** diffusion weighted imaging; **(B)** apparent diffusion coefficient map; **(C)** T_2_ weighted imaging] showed that the PI-RADS 5 lesion was located in the left transitional zone, and the mean kurtosis (MK) value was 0.562; **(D)** Color-coded parametric map showing the lesion, with a color scale bar ranging from 0 to 0.745.

A total of 84 patients were included in this study, including 39 in the TP53 mutated group (**Figure 2**) and 45 in the TP53 wild-type group (**Figure 3**). Comparison of clinical and clinicopathological characteristics between the two groups showed that only prognostic grade group (PGG) differed significantly between groups (*p* < 0.001). Specifically, compared with TP53 wild-type group, TP53 mutated group exhibited more aggressive histopathologic characteristics, with PGG ≥ 4 being the predominant category (71.80%), whereas PGG ≤ 3 was the most common in the TP53 wild-type group (73.33%). Univariate binary logistic regression analysis further demonstrated that PGG ≥ 4 was significantly associated with TP53 mutation (OR = 7.000, 95% CI: 2.678~18.295, *p* < 0.001). The details are shown in **Tables 1**, **2**.

**Table 1:** Clinical characteristics of the patients.

Variables	Mutated group (n = 39)	Wild-type group (n = 45)
Age (year)	75.21±6.03	74.64±7.20
T-PSA (ng/ml)	11.83±2.85	11.76±3.18
F-PSA (ng/ml)	1.74±0.33	1.81±0.47
F/T-PSA (ng/ml)	0.15±0.03	0.16±0.03
PI-RADS category		
2	1 (2.6%)	2 (4.4%)
3	3 (7.7%)	6 (13.3%)
4	10 (25.6%)	16 (35.6%)
5	25 (64.1%)	21 (46.7%)
Lesion Location		
TZ	17 (43.6%)	17 (37.8%)
PZ	22 (56.4%)	28 (62.2%)
PGG		
2	3 (7.7%)	22 (48.9%)
3	8 (20.5%)	11 (24.4%)
4	19 (48.7%)	7 (15.6%)
5	9 (23.1%)	5 (11.1%)
RP Surgical Margin		
Negative	32 (82.1%)	38 (84.4%)
Positive	7 (17.9%)	7 (15.6%)
RP SVI		
Negative	33 (84.6%)	40 (88.9%)
Positive	6 (15.4%)	5 (11.1%)
RP Lymphovascular Invasion		
Negative	31 (79.5%)	36 (80.0%)
Positive	8 (20.5%)	9 (20.0%)
RP Perineural Invasion		
Negative	18 (46.2%)	23 (51.1%)
Positive	21 (53.8%)	22 (48.9%)
Lymph Node Metastasis		
Negative	27 (69.2%)	34 (75.6%)
Positive	12 (30.8%)	11 (24.4%)

T-PSA, total prostate specific antigen; F-PSA, free PSA; PI-RADS, Prostate Imaging–Reporting and Data System; TZ, transition zone; PZ, peripheral zone; PGG, prognostic grade group; RP, radical prostatectomy; SVI, seminal vesicle invasion.

ECOG, Eastern Cooperative Oncology Group; IQR,Interquartile range; RAI, Radioactive iodine. Percentages calculated over 43 patients unless otherwise specified.

**Table 2:** Results of binary logistic regression analysis.

Variables	Univariable analysis		Multivariable analysis	
	OR (95% CI)	*p*	OR (95% CI)	*p*
Age (year)	1.013 (0.949~1.081)	0.698	0.919 (0.783~1.079)	0.304
T-PSA (ng/ml)	1.008 (0.874~1.164)	0.910		
F-PSA (ng/ml)	0.630 (0.205~1.933)	0.419		
F/T-PSA (ng/ml)	0.419 (0.088~1.987)	0.273		
PI-RADS category					
2	Reference		Reference	
3	0.420 (0.036~4.963)	0.491	1.072 (0.010~11.217)	0.977
4	0.420 (0.093~1.887)	0.258	7.1555 (0.209~14.069)	0.206
5	0.525 (0.197~1.399)	0.198	0.655 (0.010~7.059)	0.898
Lesion Location					
TZ	Reference		Reference	
PZ	1.273 (0.531~3.050)	0.589	1.128 (0.180~7.059)	0.898
PGG					
PGG ≤ 3	Reference		Reference	
PGG ≥ 4	7.000 (2.678~18.295)	<0.001	17.049 (3.518~79.181)	0.003
RP Surgical Margin					
Negative	Reference		Reference	
Positive	1.188 (0.377~3.744)	0.769	4.415 (0.311~62.627)	0.272
RP SVI					
Negative	Reference		Reference	
Positive	1.455 (0.407~5.196)	0.564	14.834 (0.688~31.981)	0.085
RP Lymphovascular Invasion					
Negative	Reference		Reference	
Positive	1.032 (0.355~2.999)	0.953	2.272 (0.244~21.118)	0.471
RP Perineural Invasion					
Negative	Reference		Reference	
Positive	1.220 (0.517~2.880)	0.650	1.282 (0.051~2.214)	0.258
Lymph Node Metastasis					
Negative	Reference		Reference	
Positive	1.374 (0.525~3.593)	0.517	0.466 (0.044~4.917)	0.525
MK	12.333 (4.151~36.641)	<0.001	19.329 (2.590~129.732)	0.004
MD (×10^-3^mm^2^/s)	0.423 (0.268~0.670)	<0.001	0.603 (0.228~1.596)	0.309
DDC (×10^-3^mm^2^/s)	0.266 (0.140~0.505)	<0.001	0.430 (0.159~1.168)	0.098
α	0.984 (0.788~1.227)	0.885	1.227 (0.745~2.021)	0.423
ADC (×10^-3^mm^2^/s)	0.776 (0.611~0.985)	0.038	0.722 (0.468~1.113)	0.140

T-PSA, total prostate specific antigen; F-PSA, free PSA; PI-RADS, Prostate Imaging–Reporting and Data System; TZ, transition zone; PZ, peripheral zone; PGG, prognostic grade group; RP, radical prostatectomy; SVI, seminal vesicle invasion; MK, mean kurtosis; MD, mean diffusivity; DDC, distributed diffusion coefficient; α, heterogeneity index; ADC, apparent diffusion coefficient.

### Quantitative imaging data characteristics

Among parameters of DKI, SEM and mono-exponential model, significant differences were observed between the TP53 wild-type and mutated groups in MK, MD, DDC, and ADC values (all *p* < 0.05). Compared with the wild-type group, the TP53 mutated group showed a significantly higher MK value (0.654 ± 0.057 vs. 0.551 ± 0.074, *p* < 0.001), but significantly lower MD (0.612 ± 0.072×10⁻³ mm²/s vs. 0.706 ± 0.091×10⁻³ mm²/s, *p* < 0.001), DDC (0.770 ± 0.098×10⁻³ mm²/s vs. 0.881 ± 0.126×10⁻³ mm²/s, *p* < 0.001), and ADC (0.964 ± 0.197×10⁻³ mm²/s vs. 1.052 ± 0.179×10⁻³ mm²/s, *p* = 0.034) values. The details are shown in **Table 3**.

**Table 3:** Independent samples t-test results for mono-exponential model, SEM and DKI parameters between TP53 mutated group and wild-type group.

Parameters	Mutated group	Wild-type group	F	t	*p*
MK	0.654 ± 0.057	0.551 ± 0.074	4.049	-7.155	<0.001
MD (×10^-3^mm^2^/s)	0.612 ± 0.072	0.706 ± 0.091	3.055	5.145	<0.001
DDC (×10^-3^mm^2^/s)	0.770 ± 0.098	0.881 ± 0.126	4.265	4.529	<0.001
α	0.571 ± 0.196	0.578 ± 0.196	0.040	0.143	0.886
ADC (×10^-3^mm^2^/s)	0.964 ± 0.197	1.052 ± 0.179	0.413	2.153	0.034

SEM, stretched-exponential model; DKI, diffusion kurtosis imaging; MK, mean kurtosis; MD, mean diffusivity; DDC, distributed diffusion coefficient; α, heterogeneity index; ADC, apparent diffusion coefficient.

Univariate binary logistic regression analysis showed that MK was positively associated with TP53 mutation (OR = 12.333, 95% CI: 4.151~36.641, *p* < 0.001), whereas MD (OR = 0.423, 95% CI: 0.268~0.670, *p* < 0.001), DDC (OR = 0.266, 95% CI: 0.140~0.505, *p* < 0.001), and ADC (OR = 0.776, 95% CI: 0.611~0.985, *p* = 0.038) were negatively associated with TP53 mutation. The details are shown in **Table 2**.

### Independent markers of TP53 mutation identified by multivariate binary logistic regression analysis

Due to the presence of significant multicollinearity among T-PSA (VIF = 35.192), F-PSA (VIF = 24.191), and F/T-PSA (VIF = 16.466), these variables were not included in the multivariate binary logistic regression analysis. In contrast, there was no significant multicollinearity among the other variables (all VIF < 5). Results of multivariate binary logistic regression analysis showed that PGG ≥ 4 was identified as an independent predictor of TP53 mutation (OR = 17.049, 95% CI: 3.518~79.181, *p* = 0.003). In addition, MK value also remained an independent predictor (OR = 19.329, 95% CI: 2.590~129.732, *p* = 0.004). Although MD, DDC, and ADC values were significant in univariate analysis, they lost statistical significance in the multivariate model (all *p* > 0.05). The details are shown in **Table 2**.

### Diagnostic performance of mono-exponential model, SEM, and DKI in differentiating TP53 mutated and wild-type group

ROC analysis showed that Model 4 achieved the highest AUC of 0.889 (95% CI: 0.821~0.958), followed closely by Model 1 (AUC = 0.881, 95% CI: 0.811~0.952) and Model 5 (AUC = 0.860, 95% CI: 0.784~0.937), whereas Model 2 and Model 3 yielded lower AUCs of 0.754 (95% CI: 0.649~0.859) and 0.635 (95% CI: 0.514~0.756), respectively.

Pairwise DeLong tests demonstrated that Model 1 significantly outperformed Model 2 (*p* = 0.009) and Model 3 (*p* < 0.001), while Model 4 and 5 significantly outperformed Model 2 (*p* = 0.005; *p* = 0.038) and Model 3 (*p* < 0.001; *p* = 0.001). However, no significant difference was observed between Model 1 and Model 5 (*p* = 0.273), Model 4 and Model 5 (*p* = 0.182), and the difference between Model 1 and Model 4 was also not significant (*p* = 0.518). The details are shown in **Table 4**.

**Table 4:** Results of Delong test.

Models	AUCs	Difference between AUC	Z statistic	95% CI	*p*
Model 1 vs Model 2	0.881 vs 0.754	0.127 ± 0.049	2.599	0.031 ~ 0.223	0.009
Model 1 vs Model 3	0.881 vs 0.635	0.247 ± 0.069	3.553	0.111 ~ 0.383	<0.001
Model 1 vs Model 4	0.881 vs 0.889	-0.008 ± 0.012	0.646	-0.032 ~ 0.016	0.518
Model 1 vs Model 5	0.881 vs 0.860	0.021 ± 0.019	1.097	-0.017 ~ 0.059	0.273
Model 2 vs Model 3	0.754 vs 0.635	0.120 ± 0.085	1.402	-0.048 ~ 0.287	0.161
Model 2 vs Model 4	0.754 vs 0.889	-0.135 ± 0.048	2.840	-0.228 ~ -0.042	0.005
Model 2 vs Model 5	0.754 vs 0.860	-0.106 ± 0.051	2.073	-0.206 ~ -0.006	0.038
Model 3 vs Model 4	0.635 vs 0.889	-0.255 ± 0.062	4.089	-0.377 ~ -0.133	<0.001
Model 3 vs Model 5	0.635 vs 0.860	-0.226 ± 0.070	3.226	-0.363 ~ -0.089	0.001
Model 4 vs Model 5	0.889 vs 0.860	0.029 ± 0.022	1.335	-0.014 ~ 0.072	0.182

Model 1, based on DKI (MK and MD values); Model 2, based on SEM (DDC and α values), Model 3, based on mono-exponential model (ADC value); Model 4, based on DKI + SEM + mono-exponential model; Model 5, based on MK value. AUC, area under the ROC curve; DKI, diffusion kurtosis imaging; MK, mean kurtosis; MD, mean diffusivity; SEM, stretched-exponential model; DDC, distributed diffusion coefficient; α, heterogeneity index; ADC, apparent diffusion coefficient.

**Figure 4:**
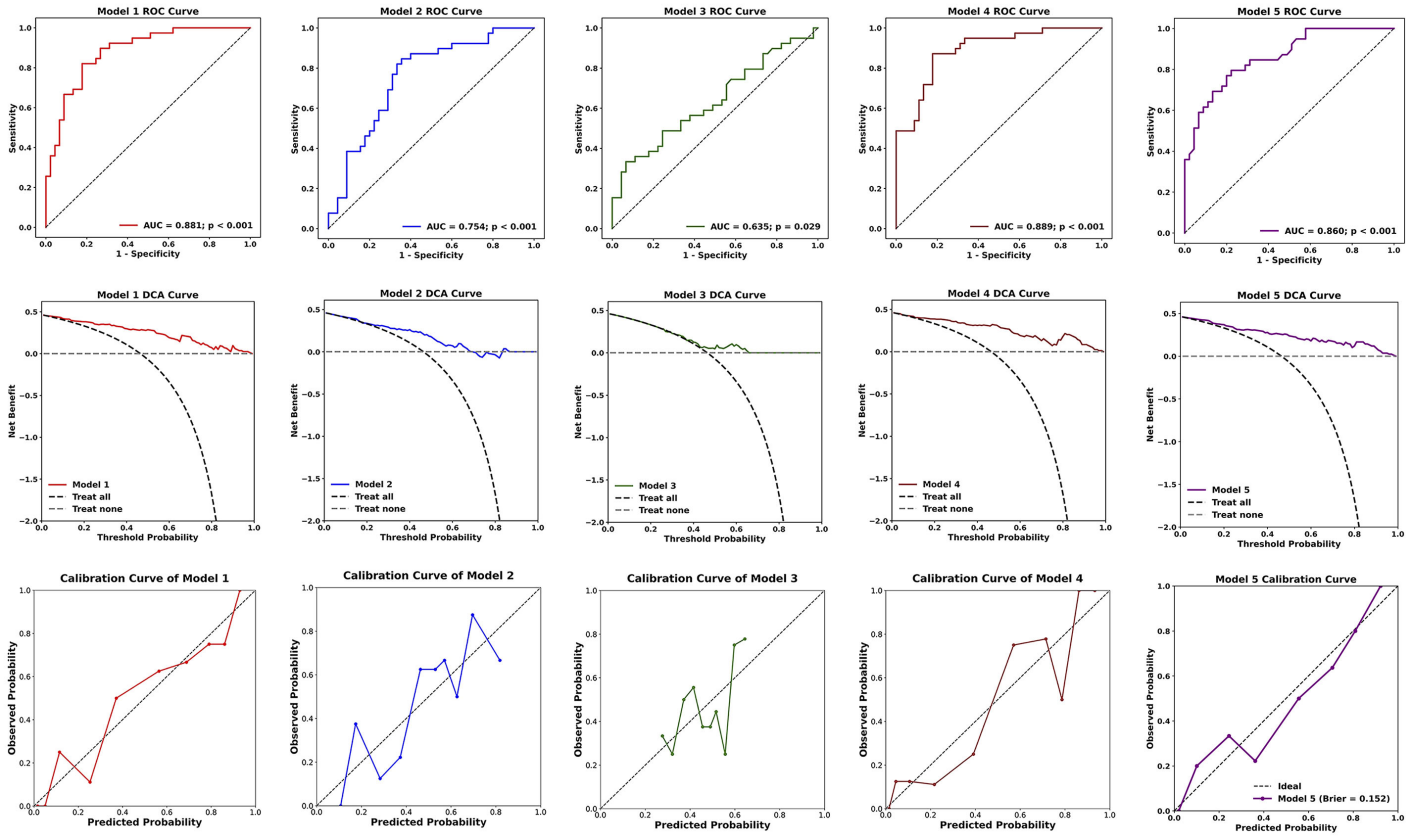
Receiver operating characteristic curve (ROC), decision curve of analysis (DCA) and calibration curve of Model 1 (based on diffusion kurtosis imaging, red curve), Model 2 (based on stretched exponential model, blue curve), Model 3 (based on mono-exponential model, green curve), Model 4 (based on diffusion kurtosis imaging, stretched exponential model and mono-exponential model, brown curve) and Model 5 (based on mean kurtosis value, violet curve).

In terms of model fit, Model 1 had the lowest AIC (77.818), followed by Model 5 (79.739), Model 4 (81.915), Model 2 (103.991), and Model 3 (115.434). Decision curve analysis further showed that Model 1, Model 4 and Model 5 consistently yielded higher net benefit than Model 2 and Model 3 across a wide range of threshold probabilities. Calibration analysis demonstrated good agreement between predicted and observed probabilities in all four models, with Hosmer–Lemeshow test P values of 0.733, 0.279, 0.540, 0.288 and 0.727 for Models 1–5, respectively. Bootstrap internal validation of Model 1 showed an apparent AUC of 0.860 and an optimism-corrected AUC of 0.858, with an optimism-corrected calibration slope of 0.979, indicating good robustness and limited overfitting. The details are shown in **Table 5**; **Figure 4**. Based on model 5, the optimal cut-off value for predicting TP53 mutation was 0.497. When the prediction probability was ≥0.497, the sensitivity and specificity for predicting TP53 mutation were 79.5% and 77.8%, respectively.

**Table 5:** Diagnostic and clinical utility performance of four predictive models for TP53 mutation in prostate cancer.

Variables	Predictive models for TP53 mutation				
	Model 1	Model 2	Model 3	Model 4	Model 5
AUC (95% CI)	0.881 (0.811–0.952)	0.754 (0.649–0.859)	0.635 (0.514–0.756)	0.889 (0.821–0.958)	0.860 (0.784~0.937)
AIC	77.818	103.991	115.434	81.915	79.739
HLχ²	5.224	9.807	6.967	9.678	5.283
HL *p* value	0.733	0.279	0.540	0.288	0.727
Brier score	0.140	0.199	0.235	0.134	0.152
Threshold probability of DCA					
0.2	0.381	0.336	0.330	0.387	0.369
0.4	0.298	0.262	0.127	0.310	0.278
0.6	0.190	0.071	0.095	0.190	0.179
0.8	0.071	-0.036	0.000	0.179	0.119

Model 1, based on DKI (MK and MD values); Model 2, based on SEM (DDC and α values), Model 3, based on mono-exponential model (ADC value); Model 4, based on DKI + SEM + mono-exponential model; Model 5, based on MK value. DKI, diffusion kurtosis imaging; MK, mean kurtosis; MD, mean diffusivity; SEM, stretched-exponential model; DDC, distributed diffusion coefficient; α, heterogeneity index; ADC, apparent diffusion coefficient; AUC, AUC, area under the ROC curve; AIC, akaike information criterion; HL, Hosmer–Lemeshow; DCA, decision curve analysis.

## Discussion

TP53 mutation is closely associated with the biological behavior of PCa and may provide important information for prognostic stratification, thereby having potential clinical value in disease management. In addition, TP53 mutation may also be involved in the development of resistance to AR pathway inhibition following ADT. Therefore, establishing a preoperative and noninvasive imaging method for characterizing TP53 mutation status would be of considerable clinical significance for risk assessment and individualized treatment planning. In the present study, Parameter MK value of DKI was identified as an independent risk factor for TP53 mutation. Moreover, the MK value-based model demonstrated good predictive performance for TP53 mutation, with an AUC of 0.860.

Results of the study showed that, compared with the TP53 wild-type group, the TP53 mutated group showed significantly lower ADC, DDC, and MD values, as well as a significantly higher MK value, which is generally consistent with previous studies (13, 16, 17, 21–23). These findings suggest that TP53 mutation PCa may possess a more complex tissue microstructure. Previous studies have shown that increased microstructural complexity and higher cellular density can further restrict water diffusion, leading to reduced diffusion-related parameters and elevated kurtosis-related parameters (24). Accordingly, our findings support the potential feasibility of using conventional DWI and its advanced diffusion models, including SEM and DKI, to assess TP53 mutation status in PCa. In addition, we observed that PGG≥4 was a risk factor for TP53 mutation in both univariable (OR = 7.000, p < 0.001) and multivariable (OR = 17.049, p = 0.003) binary logistic regression analysis, which provides additional histopathological support for the imaging differences observed in the TP53 mutated group.

It is noteworthy that although univariable binary logistic regression analysis showed that ADC, DDC, MD and MK values were all associated with TP53 mutation, only MK value remained statistically significant in the multivariable analysis, indicating that it was the only independent risk factor for TP53 mutation. This finding is similar to the results of our previous studies (25). From a biological perspective, TP53 mutation may lead to the accumulation of abnormal p53 protein and is associated with dysregulated cell-cycle control, impaired DNA repair, and altered apoptosis- and senescence-related pathways (26–29). These changes may promote uncontrolled tumor proliferation and increase both cellular density and microstructural complexity within the tumor tissue. Under such circumstances, the diffusion behavior of water molecules may deviate further from a Gaussian distribution. Compared with mono-exponential model and SEM, DKI is better suited to capture this non-Gaussian diffusion behavior, and MK value in particular may be more sensitive to the complexity of the tissue microenvironment. More specifically, ADC and DDC values mainly reflect the average magnitude of diffusion and are therefore more strongly influenced by overall cellularity and extracellular space, whereas MK value reflects the extent to which diffusion deviates from Gaussian behavior and is more sensitive to tissue disorganization, compartmental complexity, and intratumoral heterogeneity. Because TP53 mutation is biologically associated with genomic instability, aberrant proliferation, impaired apoptosis, and architectural distortion, the resultant tissue changes are likely to produce not only stronger diffusion restriction but also more complex and spatially heterogeneous diffusion patterns. This may explain why MK value retained independent significance after adjustment, whereas ADC, DDC, and MD values lost significance in the multivariable analysis.

Whether advanced diffusion models provide additional diagnostic value over the mono-exponential model in assessing PCa aggressiveness remains controversial. Some studies have reported that advanced diffusion models, such as SEM and DKI, perform similarly to the mono-exponential model in identifying Gleason patterns or clinically significant PCa, suggesting limited additional value (13, 19, 21, 22, 30). In contrast, our results showed that DKI achieved significantly better predictive performance for TP53 mutation than SEM and the mono-exponential model. Furthermore, combining parameters from DKI, SEM, and the mono-exponential model did not provide any further improvement in predictive performance. This finding is in line with some previous studies (18) and suggests that DKI may be more capable of capturing the tissue microstructural heterogeneity and complexity associated with TP53 mutation. TP53 mutation may result in the abnormal accumulation of mutant p53 protein within the nucleus and cytoplasm, thereby increasing tissue disorganization and microstructural complexity. This may partly explain why DKI outperformed SEM and the mono-exponential model in predicting TP53 mutation status in PCa. Importantly, although Model 4 achieved the numerically highest AUC, its improvement over Model 1 was minimal and not statistically significant (0.889 vs. 0.881, *p* = 0.518). In addition, Model 4 had a higher AIC than Model 1, indicating that the marginal increase in discrimination was insufficient to compensate for the increased model complexity. A likely explanation is that DDC, α, and ADC values provided partially overlapping information with that already captured by MK and MD value, so the combined model introduced more parameters without substantial incremental predictive value. Moreover, this is the reason why Model 3 yields a near-zero net benefit at higher threshold probabilities. Notably, the simplified Model 5 based on MK value alone still achieved good predictive performance and showed no significant difference from either Model 1 or Model 4, which further supports the robustness of MK value-centered modeling and the practical advantage of a more parsimonious model.

Although parameter α of SEM has been reported to differentiate benign prostatic hyperplasia from PCa, most studies have shown no consistent significant association between α and Gleason pattern (22, 31). In line with these reports, we found no significant difference in α value between the TP53 mutated and wild-type groups, suggesting that α value may be more useful for benign–malignant discrimination or certain heterogeneity features, but may have limited value in characterizing TP53 mutation–related microstructural alterations. From the perspective of SEM, α value reflects the heterogeneity of intravoxel diffusion decay rather than the absolute magnitude of diffusion restriction. Therefore, it may be more sensitive to broad architectural heterogeneity, such as differences in glandular arrangement, stromal composition, and water compartment distribution, than to molecular differences within already malignant tumors. In our cohort, both groups consisted of PCa, and TP53-related changes may not have altered the α value-defined heterogeneity pattern sufficiently or consistently. Moreover, single-slice mean ROI analysis may have averaged out focal heterogeneity, further weakening the discriminatory ability of α value.

Several limitations of this study should be acknowledged. First, this single-center retrospective study included a relatively small sample, which may limit statistical power and generalizability. Thus, the findings should be considered preliminary and require confirmation in larger multicenter prospective cohorts with independent external validation. Second, MRI-based radiomics analyses were not performed due to the limited sample size, although recent studies have highlighted their potential value (32–34). Future larger studies should integrate these approaches with quantitative diffusion parameters. Third, our analysis was based on mean quantitative parameters of the lesion, which may reduce the ability to characterize intratumoral heterogeneity and, consequently, affect the stability of predictive performance. Fourth, ROIs were delineated once by a single observer, and inter-observer reproducibility was not assessed. This may limit the reproducibility of the quantitative measurements. Fifth, although imaging–pathology matching was performed, distance-based estimation from sagittal T2WI remains approximate. Tissue deformation, sectioning differences, and limited spatial precision may have caused unavoidable mismatch, potentially affecting quantitative parameters and model performance. Finally, although the study aimed to reduce the potential impact of biopsy-related hemorrhage and inflammation, treatment-induced fibrosis, and treatment-related biological alterations on measurements of parameters of DKI and SEM, this may have introduced selection bias. Therefore, the generalizability of our findings to broader clinical populations—particularly patients who have recently undergone biopsy or received prior treatment—should be interpreted with caution.

## Conclusions

The study found that although parameter ADC value of the mono-exponential model, parameter DDC value of the SEM, and parameters MD and MK values of the DKI differed significantly between the TP53 mutated and TP53 wild-type groups, only the MK value was an independent risk factor for TP53 mutation. In terms of predictive performance, calibration consistency, and clinical net benefit, the prediction model based on MK value outperformed both the mono-exponential model and the SEM. Moreover, combining DKI with mono-exponential model and SEM did not further improve predictive efficacy. Collectively, these findings suggest that MK value may serve as a promising non-invasive imaging biomarker for preoperative prediction of TP53 mutation status in PCa.

## Data Availability

The data that support the findings of this study are available from the authors but restrictions apply to the availability of these data, which were used under license from the Second Affiliated Hospital of Dalian Medical University for the current study, and so are not publicly available. Requests to access these datasets should be directed to K-PZ, drkunpengzhou@163.com.
