# Supplementary material for: Noninvasive prediction of TP53 mutation in prostate cancer based on advanced diffusion weighted imaging
Source: Front Oncol. 2026 Jun 22;16:1854578. doi: 10.3389/fonc.2026.1854578 (PMC13333437; doi:10.3389/fonc.2026.1854578)
Supplement: Supplementary file 1 [file Table1.docx]

| **Table S1.** Acquisition Parameters of the Multiparametic MRI Protocol | | | | | | |
| --- | --- | --- | --- | --- | --- | --- |
| Sequence | Imaging plane | TR/TE (ms) | Slice/Gap (mm) | FOV (cm^2^) | Matrix | b (s/mm^2^) |
| T_2_WI | Oblique axial, Oblique coronal, sagittal | 3200-3500/95.0 | 3.0/0 | 20×20 | 256×256 | - |
| T_1_WI | Oblique axial | 767/9.6 | 3.0/0 | 24×24 | 256×224 | - |
| DWI | Oblique axial | 3500/73.0 | 3.0/0 | 24×24 | 256×224 | 0,2000 |
| DCE-MRI | Oblique axial | 3.8/1.4 | 3.0/0 | 24×24 | 256×224 |  |
| SEM | Oblique axial | 3600/88.2 | 3.0/0 | 24×24 | 256×224 | 0,25,50,75,100,150,200, 400,800,1200,2000 |
| DKI | Oblique axial | 3850/88.7 | 3.0/0 | 24×24 | 256×224 | 0, 500, 1000, 1500, 2000 |
| T_2_WI, T_2_ weighted imaging; T_1_WI, T_1_ weighted imaging; DWI, diffusion weighted imaging; DCE-MRI, dynamic contrast-enhanced imaging MRI; SEM, stretched-exponential model; DKI, diffusion kurtosis imaging; TR, repetition time; TE, echo time; FOV, field of view. | | | | | | |
